# John Cunningham (JC) Virus Encephalitis Without Progressive Multifocal Leukoencephalopathy in a Stem Cell Transplant Patient: A Case Report and Literature Review

**DOI:** 10.7759/cureus.87464

**Published:** 2025-07-07

**Authors:** Seemab Fatima, Maham Tariq, Rohab Sohail, Ridda Khattak, Muhammad Tayyeb

**Affiliations:** 1 Internal Medicine, Services Hospital Lahore, Lahore, PAK; 2 Internal Medicine, Bayhealth Hospital, Dover, USA; 3 Internal Medicine, Saint Francis Hospital, Wilmington, USA

**Keywords:** acute myeloid leukemia, encephalitis, jc virus, progressive multifocal leukoencephalopathy, stem cell

## Abstract

John Cunningham (JC) Virus (JCV) belongs to the Polyomaviridae family and is notorious for remaining latent in the kidneys and lymphoid organs of healthy populations. However, in the setting of profound immunosuppression, the virus can reactivate, leading to various manifestations depending on its location of infection, i.e., progressive multifocal leukoencephalopathy (PML), JC virus encephalopathy, or JC virus granule cell neuronopathy. Here, we present a unique case of a 28-year-old female with a notable medical history of acute myeloid leukemia (AML) status post stem cell transplant who presented to the emergency department (ED) for evaluation of her altered mental status for one week. Laboratory investigations documented the presence of JCV in both the blood and cerebrospinal fluid (CSF). Unlike the typical findings of PML, the brain imaging revealed a 4 mm T2-weighted fluid attenuated inversion recovery (T2/FLAIR) hyperintense enhancing focus in the right supratentorial region and a right anterior temporal subcortical white matter enhancement measuring up to 14 mm in size with no corresponding FLAIR abnormality suggestive of JCV encephalitis. The patient received virus-specific T cells but showed minimal to no improvement. Hence, the patient's family opted for hospice care, but the patient died subsequently. JCV encephalitis is a relatively rare condition; only a few case reports have been reported. The prognosis of JCV encephalitis is not well established and is most often lethal with high viral loads. Virus-specific T-cell therapy has not yet demonstrated the desired clinical benefit; therefore, additional data are required.

## Introduction

John Cunningham (JC) Virus Encephalitis was first described in the literature in 1958, and its causative agent, JC Virus of the Polyomaviridae family, was later isolated from the brain of a patient with Hodgkin disease in 1971 [1]. It remains latent in the kidneys and lymphoid organs without causing any active issues. JCV antibodies can be found positive in up to 86% of healthy individuals. In the setting of profound immunosuppression, this virus can reactivate and induce lytic lesions in oligodendrocytes, causing progressive multifocal leukoencephalopathy (PML). PML has a three-month mortality rate of 20-25%. Less commonly, it can also cause infection of cerebellar granule cells or cortical pyramidal neurons without classical demyelinating lesions, resulting in JCV granule cell neuronopathy or JCV Encephalitis, respectively [2]. Atypical infections have been linked to either the wild-type (archetype) JC virus or newly identified mutations in the protein-coding regions of agnoprotein or the VP1 viral capsid [3]. JCV encephalitis diagnosis also becomes somewhat challenging due to nonspecific imaging findings, making CSF JC virus PCR and clinical suspicion key to timely recognition. Here we present a case of JCV encephalitis without PML in a post-allogenic peripheral blood stem-cell transplant patient.

## Case presentation

Our patient is a 28-year-old female with a medical history of AML status post chemotherapy (cytarabine and daunorubicin) followed by an allogenic peripheral blood stem cell transplant, which was complicated by skin graft versus host disease (GVHD) and COVID-19. She presented to the emergency department (ED) with intermittent confusion and word-finding difficulty, which had been ongoing for one week. It started gradually and was progressive. Upon arrival in the ED, vital signs included blood pressure (BP) of 107/77 mmHg, heart rate (HR) of 108 beats per minute (bpm), respiratory rate (RR) of 18 breaths per minute, and temperature (Temp) of 98.4°F. The physical examination revealed a diffuse, desquamating rash over the entire skin surface. The neurologic examination was significant for diffuse encephalopathy, with no focal neurologic deficits noted. Laboratory studies showed a white blood cell count of 2.87, an absolute neutrophil count of 1,900, a hemoglobin level of 9.7 mg/dL, and a platelet count of 183,000. Further testing via PCR was conducted, which was positive for respiratory syncytial virus (RSV), COVID-19, influenza A, and JC virus (212,000 copies/ml), followed by a lumbar puncture (LP). LP was consistent with >100 million copies of JC virus and an elevated protein level (i.e., 122 mg/dL) (Table 1).

**Table 1:** Laboratory summary results JC: John Cunningham, PCR: Polymerase chain reaction, CSF: Cerebrospinal fluid, COVID-19: Coronavirus disease 2019.

Parameter	Result	Unit	Reference Range
White Blood Cell (WBC) Count	2.87	x10³/µL	4.0 – 11.0 x10³/µL
Absolute Neutrophil Count (ANC)	1,900	cells/µL	1,500 – 8,000 cells/µL
Hemoglobin	9.7	g/dL	Male: 13.5 – 17.5; Female: 12.0 – 15.5 g/dL
Platelet Count	183,000	/µL	150,000 – 450,000 /µL
RSV (Respiratory Syncytial Virus)	Positive	N/A	Negative
COVID-19	Positive	N/A	Negative
Influenza A	Positive	N/A	Negative
JC Virus (Blood PCR)	212,000	copies/mL	Negative or <500 copies/mL
JC Virus (CSF PCR)	>100,000,000	copies/mL	Negative
CSF Protein Level	122	mg/dL	15 – 45 mg/dL

**Figure 1:**
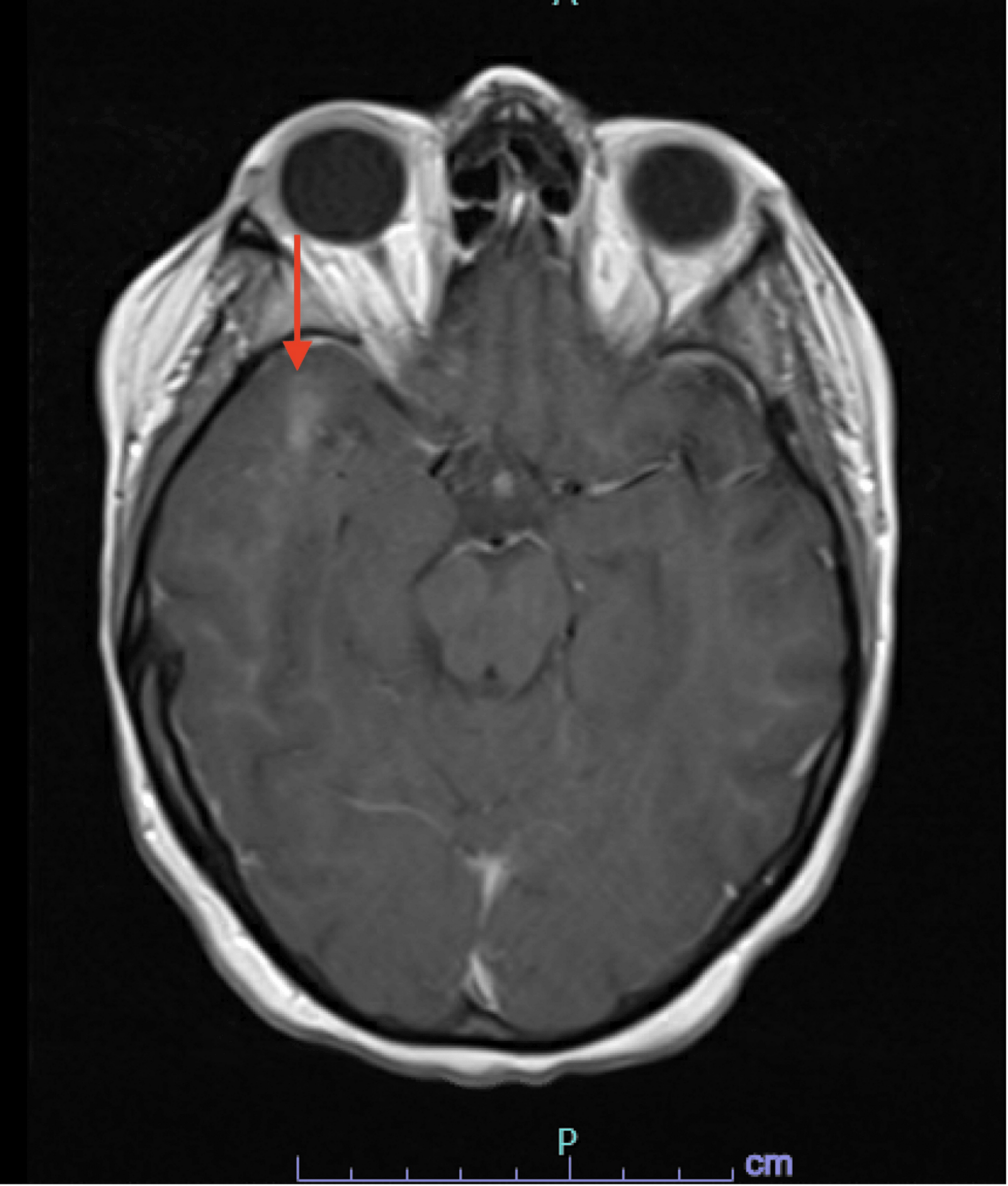
T2/FLAIR hyperintense area in anterior right temporal lobe In Figure 1, a 14 mm T2-weighted fluid attenuated inversion recovery (T2/FLAIR) hyperintense area is present in the anterior right temporal lobe (red arrow), supporting JCV encephalitis but not consistent with progressive multifocal leukoencephalopathy (PML).

**Figure 2:**
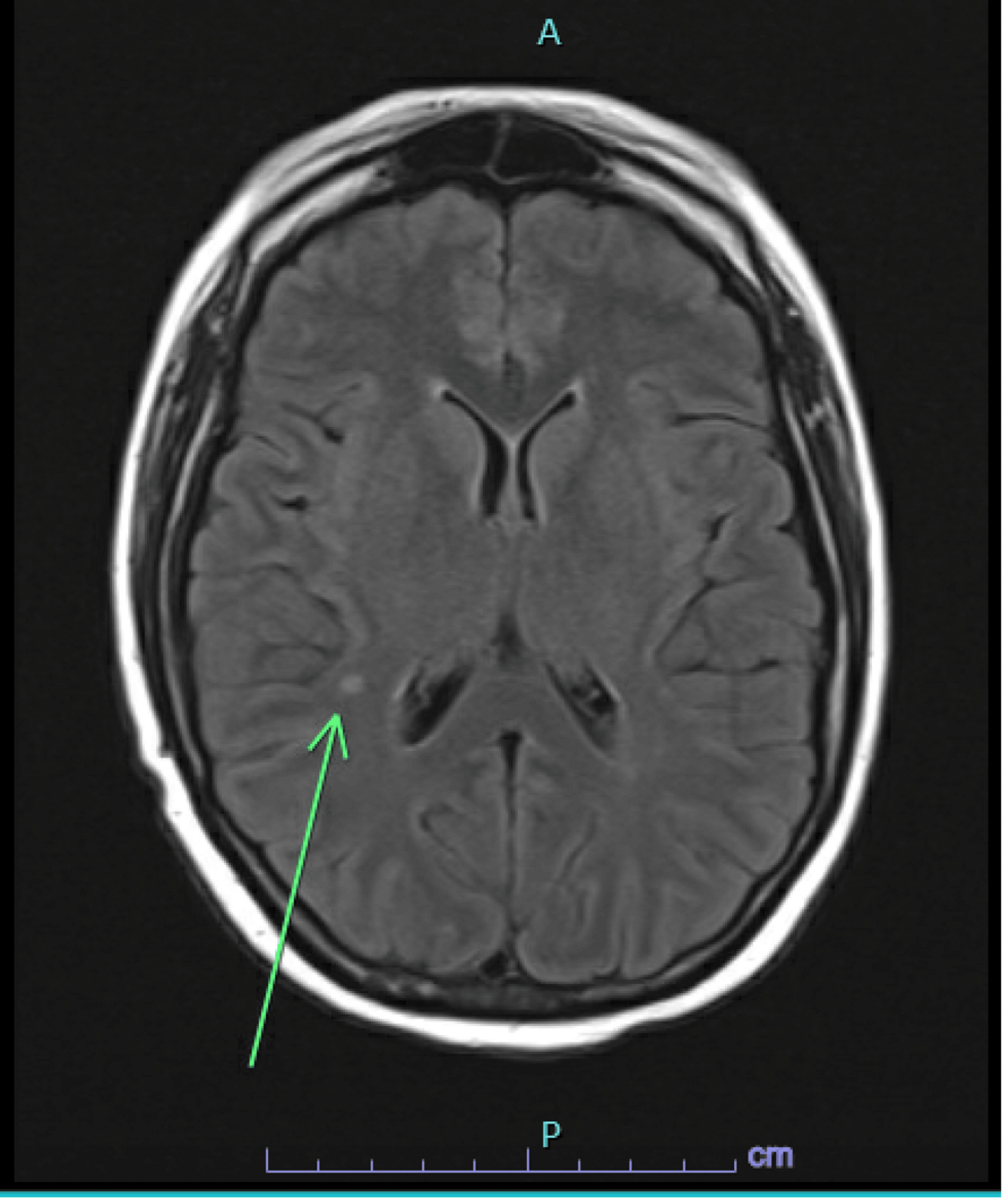
T2/FLAIR hyperintense area in right frontoparietal subcortical white matter In Figure 2, a 4 mm T2-weighted fluid attenuated inversion recovery (T2/FLAIR) hyperintense area is present in the right frontoparietal subcortical white matter (green arrow), supporting JCV encephalitis.

The remaining laboratory studies were unremarkable. Initially, a head CT was done, which was negative for acute intracranial pathology, followed by a brain MRI, which showed 4 mm T2/FLAIR hyperintense enhancing focus in the right supratentorial region and a right anterior temporal subcortical white matter enhancement measuring up to 14 mm in size with no corresponding FLAIR abnormality suggestive of JCV encephalitis instead of PML where the lesions are premodominantly present iin the periventricular region (Figures 1, 2). The patient’s mental status fluctuated throughout hospitalization. The patient received virus-specific T cell therapy after enrollment in the Clinical Trial: Third Party Viral Specific T-Cells (VSTs) for the treatment of viral infections in immunocompromised patients (NCT02532452). Treatment with virus-specific T cells significantly improved mental status with minimal to no viral load for a brief period of time. However, a few weeks later, the patient’s mental status rapidly deteriorated again. Thereafter, the patient’s family opted for hospice care.

## Discussion

JC virus (JCV) is a polyomavirus with a limited number of host cell targets, including oligodendrocytes, kidneys, urothelium, and lymphoid tissues. Although it may cause persistent, latent infection in healthy populations, it also leads to progressive multifocal leukoencephalopathy (PML), a well-documented CNS infection, in immunocompromised individuals with a debilitating prognosis. The genomic structure of JCV is composed of an early viral region that encodes for the regulatory protein and a late viral region that transcribes structural proteins (VP1, VP2, and VP3). The genetic areas are segregated by regulatory non-coding control regions (NCCR). In immunosuppressive states, NCCR serves as the hub of the most diverse genetic rearrangements, leading to an increased capacity to invade and replicate in glial cells and, hence, contributing to PML development [4]. Studies have shown a spectrum of PML pathological manifestations that corresponds to the degree of strength of adaptive immunity. 'Classic PML' manifests in the absence of substantial antiviral immunity, especially in patients with HIV infection, hematological malignancies, and patients receiving chemo-radiotherapy, stem cell transplant, and monoclonal antibody therapies [5].

Historically, the JC virus was only attributed to the infection of glial cells. Recent literature also indicates the presence of infection in non-white cellular matter within the central neuronal network, posing a diagnostic challenge for physicians regarding both clinical and radiological presentations. Some uncommon and atypical JC virus-associated diseases include JC virus granule cell neuronopathy, meningitis, and encephalopathy, primarily associated with a novel variant of JCV that has a mutation in C, the C-terminus of the VP1 capsid protein. However, wild-type JCV has also been isolated from such cases [3]. JCV encephalopathy is a rare condition, with only a few cases reported. Wuthrich et al. reported a case of JCV encephalopathy that developed aphasia and progressive cognitive decline in the absence of focal neurologic deficits after chemotherapy for non-small cell lung cancer [6].

The diagnostic criteria for every JCV-associated CNS infection include positive JCV DNA polymerase chain reaction (PCR), MRI features, and brain biopsy. PCR amplification of JCV DNA of cerebrospinal fluid is a pivotal part of diagnosis, with the capacity to detect JCV DNA even in patients with low viral load. Its specificity is around 95-100%, while the sensitivity increases from 70% to 100% as the disease progresses to the late stages [7,8]. MRI of the brain gives characteristic findings of various JC infections. Classical PML findings include asymmetric, sharply demarcated periventricular, non-enhancing multifocal and, rarely, unifocal subcortical and juxtacortical lesions, which are hyperintense on T2-weighted images and fluid-attenuated inversion recovery (FLAIR) images, with corresponding areas of hypointensity on T1-weighted images [4]. On the contrary, MRI in PML with Immune Reconstitution Syndrome (IRIS) typically shows contrast enhancement, edema, and a mass effect -features indicative of inflammation and damage to the blood-brain barrier. A specific MRI feature of natalizumab-associated PML includes perilesional contrast enhancement on T2-weighted imaging, often remarked as a 'Milky Way' appearance. JCV granule cell neuronopathy is evident on radiology as cerebellar atrophy, while JCV encephalopathy can present with or without non-enhancing brain cortical lesions. JCV meningitis lacks the characteristic radiological features. However, it can show dilatation of the ventricles [9].

Brain biopsy and histopathology are critical to the definitive diagnosis of JVC infections. Classical PML has a histological triad of bizarrely enlarged astrocytes with irregular nuclei, demyelinating lesions at the grey-white junction, and enlarged, hyperchromatic nuclei of oligodendrocytes, accompanied by inflammatory infiltrates in PML-IRIS [10]. The atypical JCV infections primarily exhibit lytic lesions under the microscope, more pronounced in cerebellar granule cells and cortical pyramidal cells, respectively, in JCV granule cell neuronopathy and encephalopathy [3,11]. The course of positive laboratory findings and MRI indicators in JCV infections often remains unrelated and unparalleled. There have been reported symptomatic cases with early atypical brain lesions in radiological studies with negative laboratory workup and vice versa, requiring repeated imaging and PCR tests over time for confirmation [11].

Regrettably, there's no specific treatment for JCV-associated infections, including PML, JCV encephalopathy, and JCV granule cell neuronopathy. Restoring the host adaptive immune response remains the mainstay of management, which includes discontinuation or reduction of immunosuppressive agents, such as glucocorticoids or calcineurin inhibitors, in transplant patients [12,13]. The advantage of this treatment is uncertain, and it is clear that there is a higher likelihood of rejection in patients with organ transplants or relapse in patients with inflammatory conditions. Nonetheless, some patients with hematologic malignancies may not be able to achieve immune reconstitution, even after discontinuation of immunosuppressive therapy, due to long-term depletion of immune cells or impaired bone marrow function. Several pharmacological options, including cytarabine, cidofovir, topotecan, and maraviroc, have been tried to treat JCV infection; however, none of these medications have proven to have a net clinical benefit in randomized clinical trials or prospective studies. Therefore, these drugs are not considered adequate for the treatment of PML [14,15]. An investigational treatment for JCV infection involves using virus-specific cytotoxic T cells generated from the patient's cells (autologous) or allogenic third-party donors. This approach has shown promise in treating JCV infection, particularly in the context of PML. Berzero et al. reported the results of a cohort of nine HIV-negative patients with immune suppression due to hematologic malignancies or congenital immune deficiencies who developed PML [16]. They received JCV-specific T cells generated ex vivo or banked JCV-specific T cells. After a median follow-up of 39 months, four of the nine patients had neurologic improvement. They survived with a mild or minimal residual disability; one survived with severe disability, and four died [16]. No treatment-related adverse events were noted. Our patient also received JCV-specific cytotoxic T cells, initially obtained from hematopoietic stem cell transplant (HSCT) donor cells, which resulted in transient clinical improvement; however, the clinical symptoms worsened afterward.

There is no significant data regarding the prognosis of JCV encephalitis without PML. However, the overall median survival of patients with PML (without HIV infection) is three months [17]. A retrospective study that included 107 PML cases associated with hematologic malignancies did show that patients who received hematopoietic HSCT had lower mortality (56% versus 88%) and more prolonged median survival (eight months vs. two months) as compared to the patients who received chemotherapy or immunotherapy [18]. JCV levels may have a prognostic value in patients with JCV infection, but this has not been proven yet. Yiannoutsus et al. reported the results of a small study in which a low JCV burden in CSF (50 to 100 copies/mL) was associated with more prolonged survival than patients with a high JCV burden [19].

Our patient is unique in that she had a subcentimeter unifocal subcortical lesion that remained stable on several repeated MRI scans and didn't have typical clinical features and imaging expected for PML, which was quite unusual for JCV infection findings, as discussed above. This case highlights that a poor prognosis is highly associated with the viral load copies of JCV. Henceforth, signifying the importance of early detection, limited disease progression, and management, with reduction or discontinuation of immunosuppressive therapies being the core of an effective management strategy. Monitoring of JCV DNA in CSF is crucial to assessing the patient's therapeutic response. It also emphasizes that virus-specific T-cell therapy has yet to show clinical benefits, and more data is required.

## Conclusions

This rare case of JC virus encephalitis without PML in a stem cell transplant recipient highlights the importance of early recognition, diagnosis via CSF PCR, brain imaging, and prompt immune-restorative interventions. Despite virus-specific T-cell therapy, the prognosis remains poor, highlighting the urgent need for further research into effective treatments for JCV encephalitis.
